# RUPTURED SPLENIC ABSCESS WITH PNEUMOPERITONEUM SECONDARY TO CHRONIC PYELONEPHRITIS

**DOI:** 10.51894/001c.122882

**Published:** 2024-08-30

**Authors:** Erica Roach, Hutton White, Jacob Schaub, Kyle Moehlin, Michael Kopek, Tarik Wasfie

**Affiliations:** 1 College of Osteopathic Medicine Michigan State University

## 30

### INTRODUCTION

Splenic abscesses are relatively uncommon occurrences with autopsy studies estimating the incidence to be between 0.2% to 0.07%. However, the mortality rate from splenic abscesses remains high, especially for immunocompromised patients. The best course of treatment is still unclear, but current literature demonstrates that early detection and source control have been shown to improve outcomes. There are several management options available, including treatment with antibiotics, percutaneous and surgical drainage. However, surgical intervention is currently considered the standard of care due to its effectiveness compared to percutaneous drainage, which has a high inefficacy rate ranging from 14.3% to 75% (Pena-Ros, et al.). Additionally, splenic abscesses pose a diagnostic challenge due to their non-specific presentation. Recent advancements in imaging modalities, such as CT scans and ultrasonography (US), have led to higher detection rates. The United States, in particular, has been instrumental in detecting splenic abscesses, due to its affordability, safety, accessibility, and precision.

### CASE DESCRIPTION

We present a unique case of a woman with end-stage renal disease on hemodialysis, who is otherwise immunocompetent presenting with septic shock from a perforated splenic abscess and pneumoperitoneum.

### DISCUSSION

Splenic abscesses have a bimodal age distribution with peaks at 30 and 60 years of age. About two-thirds of splenic abscesses in adults are solitary, while one-third are multiple. Very rarely, a ruptured splenic abscess can cause pneumoperitoneum, and this may require the abscess to have formed secondary to a gas-forming organism. Pneumoperitoneum typically occurs secondary to perforation of a hollow organ, such as gastric perforation, perforated diverticulitis/appendicitis, perforated bowel malignancy, or perforation of a strangulated intestinal obstruction. Therefore, when evaluating a patient with pneumoperitoneum, it is crucial to consider the possibility of a perforated splenic abscess in the differential diagnosis.

